# Gene Silencing Mediated by Endogenous MicroRNAs under Heat Stress Conditions in Mammalian Cells

**DOI:** 10.1371/journal.pone.0103130

**Published:** 2014-07-28

**Authors:** Masashi Fukuoka, Mariko Yoshida, Akiko Eda, Masaki Takahashi, Hirohiko Hohjoh

**Affiliations:** Department of Molecular Pharmacology, National Institute of Neuroscience, NCNP, Kodaira, Tokyo, Japan; University of Torino, Italy

## Abstract

Heat shock, sudden change in temperature, triggers various responses in cells for protecting the cells from such a severe circumstance. Here we investigated gene silencing mediated by endogenous microRNAs (miRNAs) in mammalian cells exposed to a mild hyperthermia, by means of miRNA activity assay using a luciferase reporter gene as well as miRNA expression analysis using a DNA microarray. Our findings indicated that the gene silencing activities involving miRNAs were enhanced without increasing in their expression levels under heat-stress conditions. Additionally, the gene silencing activity appeared to be independent of the cytoprotective action involving heat shock proteins that are immediately activated in heat-shocked cells and that function as molecular chaperons for restoring heat-denatured proteins to normal proteins. Our current findings suggested the possibility that gene silencing involving endogenous miRNAs might play a subsidiary role in heat-shocked cells for an aggressive inhibition of the expression of heat-denatured proteins.

## Introduction

MicroRNAs (miRNAs) are 21∼23-nucleotide-long small non-coding RNAs that are processed from longer (initial) transcripts forming stem-loop structure by digestion with RNase III enzymes, Drosha in the nucleus and Dicer in the cytoplasm. The processed, or matured, miRNA is incorporated into the RNA-induced silencing complex (RISC) and functions as a mediator in gene silencing (review articles [1]–[3]). MicroRNAs play essential roles in gene regulation by inhibiting translation of messenger RNAs (mRNAs) that are partially complementary to the miRNAs, and by digestion of mRNAs that are nearly complementary to the miRNAs, or by RNA interference (RNAi), during various vital function and phenomenon such as cell proliferation, differentiation, development and senescence [1]–[6].

Hundreds of miRNA genes have been found in animals and plants [see the microRNA database (miRBase): http://www.mirbase.org/index.shtml]. Most of miRNA genes appear to be expressed by RNA polymerase II [7], and expression profile analyses of miRNAs provide us with useful information in understanding complex gene regulation involving miRNAs as well as in characterizing miRNAs themselves. Many expression studies on miRNAs in various tissues and cells have been carried out, thereby revealing tissue- and stage-specific expression of miRNAs [8]–[14]. In mammals, it has been found that a major small RNA class transition from retrotransposon-derived small interfering RNAs (siRNAs) and Piwi-interacting RNAs (piRNAs) to zygotically expressed miRNAs occurs during pre-implantation development [15], and tissue- or organ-specific expression patterns of miRNAs are generated thereafter. MicroRNAs appear to participate in various vital functions via gene regulation involving their gene silencing (review articles [16]–[18]), and some of them may be capable of becoming useful molecular markers that reflect biological changes and functions.

Heat shock, sudden change in temperature, is an external stress; and cells that are subjected to heat shock immediately respond to such an environmental stress for protecting themselves and for maintenance of homeostasis. A major response to heat shock is the expression of a certain set of proteins, referred to as heat-shock proteins (HSPs), and HSPs play important roles in cells under heat stress conditions (review articles [19]–[22]). Of the HSP functions, the role of HSPs as a molecular chaperon appears to be particularly important, and misfolded proteins that are caused by heat shock may be helped for refolding into their correct shapes by association with HSPs. A recent study suggested that HSP70 and HSP90 likely associated with Ago2 protein that is a major component of RISC, and would participate in loading of small RNAs into RISCs [23]; the findings lead us to the possibility that there may be some sort of relationship between heat shock and gene silencing.

In this study, we investigated gene silencing mediated by endogenous miRNAs in mammalian cells that were subjected to a mild hyperthermia, and our findings suggested that the activity of gene silencing involving many miRNAs was increased without increasing in their expression levels under such heat-stress conditions.

## Materials and Methods

### Cell culture

HeLa cells were grown in Dulbecco's modified Eagle's medium (DMEM) (Wako, Osaka, Japan) supplemented with 10% fetal bovine serum (Invitrogen, Carlsbad, CA, USA), 100 units/ml penicillin, and 100 µg/ml streptomycin (Wako) at 37°C in 5% CO_2_ humidified chamber. For heat shock, cells were subjected to a mild hyperthermia at 40°C for 12 h in 5% CO_2_ humidified chamber.

### DNA and RNA oligonucleotides

DNA oligonucleotides and small RNA duplexes used in this study were synthesized by and purchased from Life Technologies (Carlsbad, CA, USA) and Sigma-Aldrich (St Louis, MO, USA), respectively.

### Inhibitors used in this study

Geldanamycin (LC Laboratories, Woburn, MA, USA), LY294002 (Cell Signaling Technology, Danvers, MA, USA), PD98059 (Cell Signaling Technology) and SB203580 (Cayman Chemical, Ann Arbor, MI, USA) used as HSP90-, PI3K-, MEK- and p38/MAPK-inhibitors, respectively, were dissolved in dimethyl sulfoxide (DMSO) (Sigma-Aldrich).

### Construction of reporter genes

To examine the effects of endogenous miRNAs on gene silencing against their targets, we constructed reporter genes with the psiCHECK-2 vector (Promega, Fitchburg, WI, USA) (Fig. S1): the *Renilla luciferase* gene carrying the complementary (target) sequence of miRNAs in the 3′ untranslated region was constructed as described previously [24]. The target sequences synthesized in the construction are indicated in Table S1.

### Transfection of reporter plasmids and reporter assay

The day before transfection, HeLa cells were trypsinized, diluted with medium without antibiotics, and seeded onto 96-well culture plates (0.25–0.5×10^4^ cells/well). The psiCHECK-2-backbone plasmids (20 ng/well) were introduced into cells by using a Lipofectamine2000 transfection reagent (Life Technologies) according to the manufacturer's instructions. After 18 h-incubation, the cells were exposed to a mild hyperthermia at 40°C for 12 h. After heat treatment, cells were lysed in passive lysis buffer (Promega), and the expression of *Photinus* and *Renilla* luciferases were examined by a Dual-Luciferase Reporter Assay system (Promega) according to the manufacturer's instructions. The luminescent signals were measured by means of a Fusion Universal Microplate Analyzer (Perkin Elmer, Waltham, MA, USA).

The pGL4 vectors carrying various response elements (Promega) (40 ng/well) were introduced together with the phRL-TK plasmid (10 ng/well, Promega) into HeLa cells, and the cells were subjected to a mild hyperthermia at 40°C followed by dual luciferase assay as described above. The pGL4 vectors used in this study were as follows (abbreviated name used in this study):

pGL4-27 [*luc2P*/minP/Hygro] (minP), pGL4-41 [*luc2P*/HSE/Hygro] (HSE), pGL4.29 [*luc2P*/CRE/Hygro] (CRE), pGL4.32 [*luc2P*/NF-κB-RE/Hygro] (NF-κB-RE), pGL4.33 [*luc2P*/SRE/Hygro] (SRE), pGL4.34 [*luc2P*/SRF-RE/Hygro] (SRF-RE).

### RNAi knockdown

To silence the *HSF1* gene, two siRNAs were designed and chemically synthesized. The synthetic siRNAs (30 nM, final concentration) were transfected into cells, using a Lipofectamine2000 transfection reagent (Life Technologies) according to the manufacturer's instructions. The treated cells were incubated for 48 h and subjected to subsequent examinations. The sequences of the siRNAs were as follows:

siHSF1 #1;

Sense-strand; 5′ - GGCCAUGAAGCAUGAGAAUUU-3′

Antisense-strand; 5′ - AUUCUCAUGCUUCAUGGCCUU-3′

siHSF1 #2;

Sense-strand; 5′ - GUUGUUCAUAGUCAGAAUUUU-3′

Antisense-strand; 5′ - AAUUCUGACUAUGAACAACUU-3′

### Total RNA preparation and reverse transcription (RT)-(real time)-polymerase chain reaction (PCR)

Total RNAs were extracted from cells using a TRI Reagent (MRC, Cincinnati, OH, USA) according to the manufacturer's instructions. Complementary DNA (cDNA) synthesis for matured miRNAs was carried out using a TaqMan MicroRNA Reverse Transcription Kit (Life Technologies) with TaqMan MicroRNA Assays (Life Technologies), and cDNA synthesis for primary miRNAs was performed using Random hexamers (Life Technologies) and a Superscript III reverse transcriptase (Life Technologies). Quantitative PCR (qPCR) was carried out using the AB7300 Real Time PCR System (Life Technologies) with a FastStart Universal Probe Master Mix (Roche) together with TaqMan MicroRNA Assays (Life Technologies) according to the manufacturer's instructions. The TaqMan MicroRNA and Pri-miRNA Assays used were as follows (Assay IDs are indicated in parentheses):

*hsa-miR-29a (2112), hsa-miR-221 (524), hsa-miR-193a-3p (2250), hsa-miR-135b (2261), hsa-miR-186 (2285), hsa-miR-296 (527), hsa-miR-125b (449), hsa-miR-92 (430), hsa-miR-16 (391), hsa-miR-19b (396), hsa-miR-26a (405), hsa-miR-29b (413), snoU6 RNA (1232), hsa-miR-494 (2365), and hsa-miR-494 (Hs04225959; Pri-miRNA assay)*

To examine endogenous gene expression, total RNAs were prepared from cell extract containing 0.1 ng (1.8×10^8^ copies) of λpolyA^+^ RNA-A [External Standard Kit (λpolyA) for qPCR; TAKARA BIO, Otsu, Shiga, Japan] that was added as an external control, and subjected to cDNA synthesis using oligo (dT)_15_ primers (Promega) and a Superscript III reverse transcriptase (Life Technologies) according to the manufacturer's instructions. The resultant cDNAs were subjected to qPCR analysis using the AB 7300 Real Time PCR System (Applied Biosystems) with a FastStart Universal SYBR Green Master (Roche) and Perfect Real Time primers (TAKARA BIO) according to the manufacturer's instructions. The Perfect Real Time primers used were as follows (TAKARA BIO primer-set IDs):

*COL4A1* (HA156982), *COL4A2* (HA174126), *CABIN1* (HA183487), *DNMT1* (HA002770), *DICER1* (HA174786), *LAMC1* (HA083467) and *GAPDH* (HA067812).

The level of λpolyA^+^ RNA-A as an external control was examined by qPCR using the AB 7300 Real Time PCR System (Applied Biosystems) with a FastStart Universal SYBR Green Master (Roche) and an External Standard Kit (λpolyA) for qPCR (TAKARA) according to the manufacturer's instructions.

### Western blot analysis

Cells were washed with PBS (Wako) and lysed in RIPA buffer [25 mM Tris-HCl (pH7.6), 0.1% SDS, 150 mM NaCl, 1% sodium deoxycholate, 0.5% NP-40] containing 1 x protease inhibitor cocktail (Protease Inhibitor Cocktail Tablets; Roche Diagnostics, Basel, Switzerland). The lysate was incubated on ice for 20 min, sonicated (10 sec, 3 times) and centrifuged at 14,000×g for 10 min at 4°C. The resultant supernatant (cell lysate) was collected. Protein concentration of the cell lysate was measured by a protein quantification kit-wide range (DOJINDO, Mashiki-town, Kumamoto, Japan). Equal amounts of protein (∼40 µg) were mixed with 2 x sample buffer (125 mM Tris-HCl pH6.8, 2% glycerol, 4% SDS, 0.02% bromophenol blue, 10% beta-mercaptoethanol) and boiled for 5 min. The protein samples were electrophoretically separated on 8% or 10% SDS-polyacrylamide gels (SDS-PAGE), and blotted onto polyvinylidene fluoride membranes (Immobilon P; Millipore, Billerica, MA, USA). The membranes were incubated for 1 h in blocking buffer (TBS-T containing 5% skim milk) and then with diluted primary antibodies at 4°C overnight or at room temperature for 1 h. After incubation, the membranes were washed in TBS-T, and incubated with 1/5000 diluted horseradish peroxidase-conjugated goat anti-mouse IgG (Sigma-Aldrich) or goat anti-rabbit IgG (Sigma-Aldrich) for 30 min at room temperature. Antigen-antibody complexes were visualized using an Immobilon western (Millipore) according to the manufacturer's instructions. The primary antibodies used in Western blotting and their product IDs and dilution ratios in parentheses were as follows:

Anti-Akt (#9272, 1/1000), anti-HSF1 (#4356, 1/1000), anti-DICER (#3363, 1/1000), and anti-AGO2 (#2897, 1/1000) were purchased from Cell Signaling Technology. Anti-HSP90AB1 (SAB4300541, 1/1000), anti-HSP70 (SAB2700846, 1/1000), anti-HSP27 (SAB4501457, 1/1000), and anti-α-Tubulin (F2168, 1/5000) were purchased from Sigma-Aldrich.

### DNA chip analysis

*Genopal* ^−^MICH07 DNA chips (Mitsubishi Rayon, Tokyo, Japan) were used for detection of human miRNAs as described previously [6], [11]. DNA oligonucleotide probes for 188 miRNAs are installed on the DNA chip, and the list of the detectable miRNAs is available at the following address: http://www.mrc.co.jp/genome/pdf/products/micro_rna_mich07.pdf.

### Accession number

The Gene Expression Omnibus (GEO) accession number of the miRNA expression data used in this study is GSE53745.

## Results

### Enhancement of gene silencing involving miRNAs after heat shock

To examine gene silencing involving a number of miRNAs at one time, or the profile of gene silencing, we constructed 143 reporter plasmids that carried the complementary sequences of various human matured miRNAs, which were relatively initially-identified and randomly-selected, in the 3′-untranslated region of the *Renilla luciferase* reporter gene based on the psiCHECK-2 vector used as a backbone plasmid (Fig. S1 and Table S1). The reporter plasmids allow for identification of active miRNAs that are working as mediators in RISCs in cells; and analyses with the reporter plasmids together with expression profile analyses of miRNAs by a DNA microarray that can detect the physical presence of miRNAs containing active and inactive miRNAs may provide us with valuable information on the regulation of gene silencing, for example a possible regulation of functional miRNAs in RISCs under different conditions.

**Figure 1:**
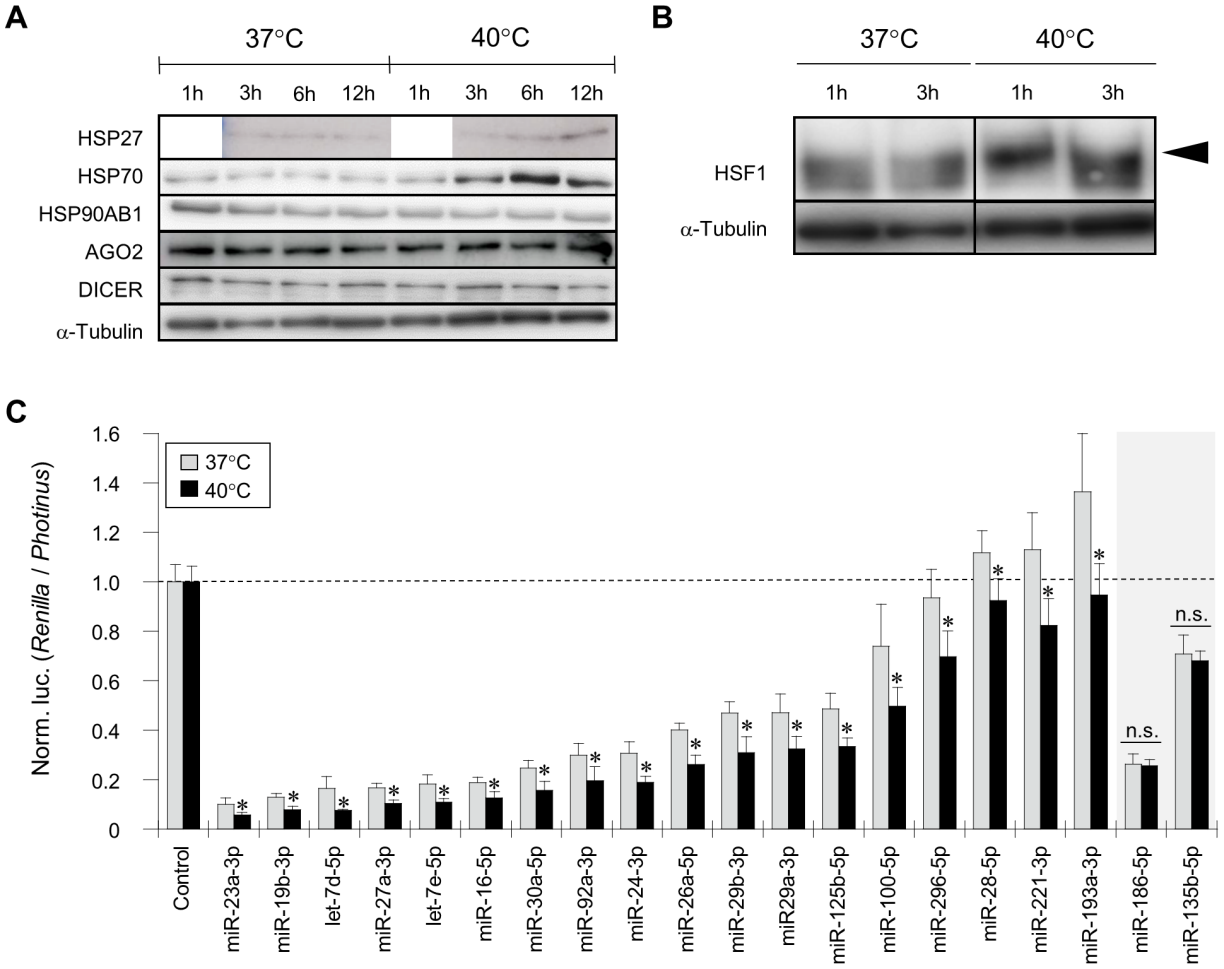
Heat shock responses and gene silencing mediated by endogenous miRNAs in HeLa cells exposed to a mild hyperthermia. (**A**) Western blot analysis. HeLa cells were exposed to a mild hyperthermia (40°C) as well as normal temperature (37°C). After starting incubation at 40°C, cell extracts were prepared at indicated time (h: hours), and examined by Western blotting using antibodies directed against the proteins indicated. Alpha-tubulin was examined as an internal loading control. (**B**) Early response of HSF1 to heat shock. An acute phosphorylation of HSF1 immediately after heat shock was examined by Western blotting using the same samples as in A. An arrowhead indicates phosphorylated HSF1. (**C**) Gene silencing profiles involving endogenous miRNAs. The psiCHECK-2 plasmids (Fig. S1) that carried the complementary sequences of target miRNAs (indicated) were transfected into HeLa cells cultured in 96-well culture plates, and the transfection was duplicated. After 6 h-incubation at 37°C, the culture plates, that is transfected cells, were divided into two groups; and a group was further incubated at 37°C (gray bars) for 12 h, and the other group was cultured at 40°C (black bars) for 12 h. After incubation, dual-luciferase assay was carried out. The activity of the *Renilla* luciferase (target) was normalized to that of the *Photinus* luciferase, and further normalized to the data obtained from the cells that were transfected with the psiCHECK-2 empty plasmid as a control. The data are averages of four independent experiments and error bars represent standard deviations. Significant difference between the 37°C and 40°C data was analyzed by Student's t-test (two-tailed; * *p*<0.05). n.s., no significant difference.

Our current study investigated gene silencing involving endogenous miRNAs under heat-stress conditions by using the constructed reporter plasmids and a DNA microarray for miRNAs. HeLa cells were subjected to a mild hyperthermia at 40°C for 12 h, and the cells appeared to respond to the heat stress: immediate phosphorylation of heat shock transcription factor 1 (HSF1) and marked increase in heat shock protein 27 (HSP27) and 70 (HSP70) after the treatment were detected by Western blotting (Fig. 1A and B). Under such a heat-stress or heat-shocked condition, gene silencing activities involving miRNAs were investigated by means of the constructed reporter plasmids (Figs. 1C and S2). Interestingly, apparent suppression efficiencies involving a number of miRNAs in heat-shocked HeLa cells appeared to be enhanced relative to those in naïve cells (under a normal condition, at 37°C); as a matter of course, there were miRNAs (*e.g.*, miR-135b-5p and miR-186-5p) that conferred no significant difference in suppression efficiency between the heat-shocked and naïve cells, as well as miRNAs associated with the reduction of the efficiencies. As a possible interpretation of the results, change of miRNA expression correlated to the alteration of gene silencing activity might have happened in heat-shocked cells.

As additional data, the expression levels of the *luciferase* reporter genes appeared to be slightly reduced in heat-shocked cells (Fig. S3), suggesting the possibility that the promoters of the reporter genes might be influenced by heat shock. In this study, gene silencing efficiencies involving endogenous miRNAs were examined and compared based on normalized suppression ratios of target reporter genes under each temperature condition; hence little or no biased assessment might occur in the comparison. Additionally, even though the reduction of gene expression under heat-stress conditions might influence the suppression efficiency of miRNA-mediated gene silencing, it cannot fully explain the enhancement of the suppression efficiency, in light of exceptional instances that showed little or no difference in the suppression efficiency between heat-shocked and naïve cells (Figs. 1C and S2).

### Expression profile of miRNAs in heat-shocked cells

**Figure 2:**
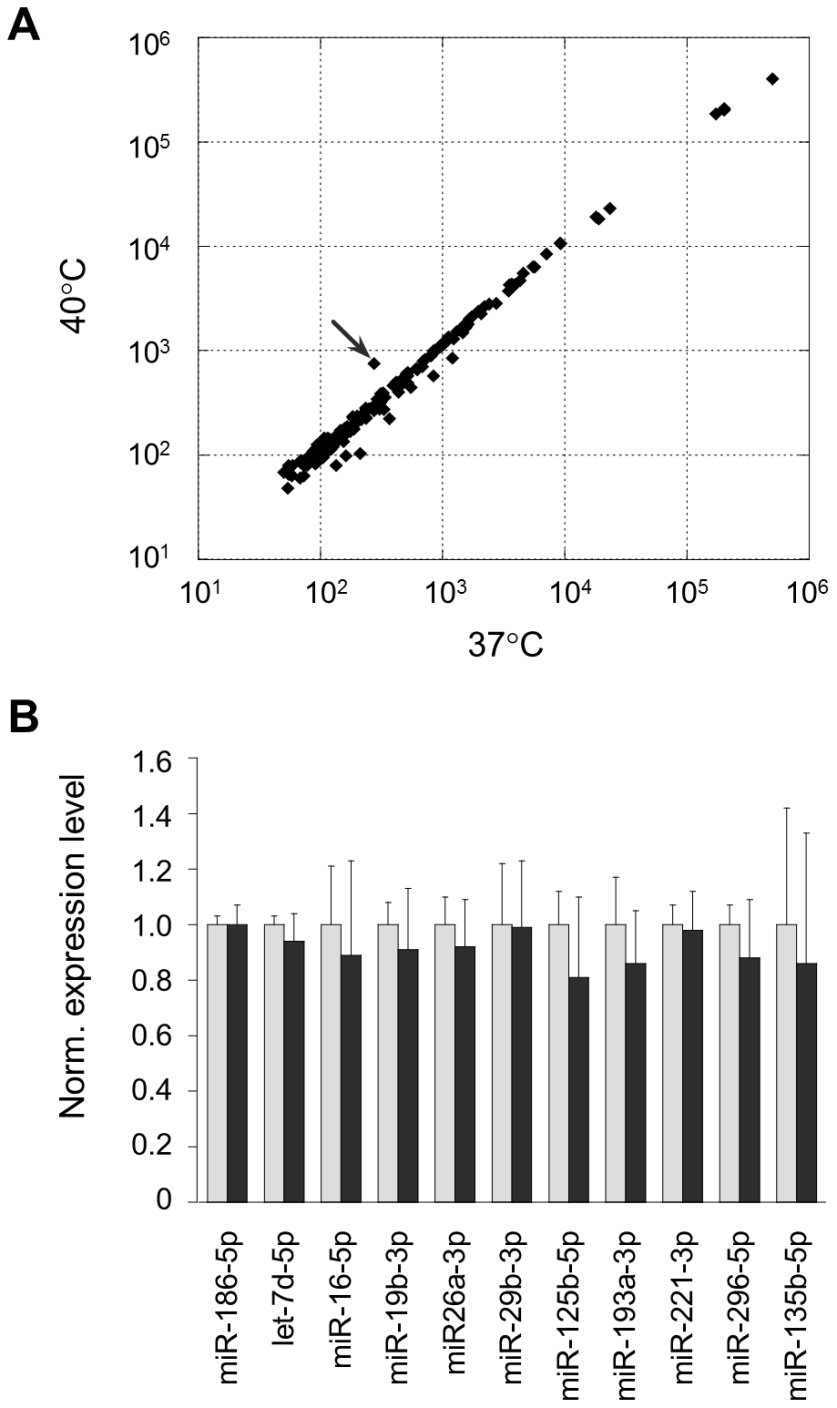
Expression profiles of miRNAs under normal and heat stress conditions. (**A**) Expression profiles of miRNAs in HeLa cells that were exposed to a mild hyperthermia (40°C) as well as normal temperature (37°C) for 12 h were examined by means of the *Genopal*-MICH07 DNA chip. The expression profile data were compared to each other by scatter-plot graphs. The data were represented by hybridization signal intensities and indicated by arbitrary units. An arrow indicates miR-494 which looked like being upregulated in heat-shocked cells. The DNA chip analysis was duplicated with different samples and similar results were obtained. (**B**) RT-qPCR. To further confirm the expression data, RT-qPCR was carried out using newly prepared samples. The expression data were analyzed by the delta-delta Ct method using the data of miR-186-5p as a reference, which showed no significant difference in the gene silencing activity between naïve and heat-shocked cells (Fig. 1C), and further normalized to the data obtained from the normal temperature (37°C). The data are averages of three determinations, and error bars represent standard deviations. No significant difference between the 37°C (gray bars) and 40°C (black bars) data was detected by Student's t-test (two-tailed, *p*<0.05).

To investigate the expression level of miRNAs in heat-shocked and naïve HeLa cells, we performed expression profile analyses of miRNAs by using *Genopal*–MICH07 DNA chips [6], [11]. Duplicated DNA chip analyses with independently prepared RNA samples exhibited reproducible results; and the results, contrary to our expectation, revealed no significant difference in the expression profiles between the heat-shocked and naïve HeLa cells except for miR-494 in heat-shocked cells (Fig. 2A). We confirmed the data by RT-qPCR and obtained consistent results (Fig. 2B). As for miR-494, we did not know why, but little or no difference in its level was detected by RT-qPCR with different samples prepared from different cells (Fig. S4A). In addition, no significant difference in gene silencing activity involving miR-494 between naïve and heat-shocked cells was also seen (Fig. S4B).

Together with the data of gene silencing profile (Figs. 1C and S2), the findings suggested the possibility that the suppression efficiency of miRNA-mediated gene silencing might be capable of changing without increasing and decreasing in the level of miRNAs in heat-shocked cells.

### Expression of AGO2, DICER, and endogenous genes under heat-stress conditions

We tried to find the factor(s) that is implicated in changing the suppression efficiency of miRNA-mediated gene silencing under heat-stress conditions. Since heat stress most likely influences existing proteins and gene expression in cells, we first examined AGO2 and DICER, both of which are key proteins in gene silencing, by Western blotting. As shown in Fig. 1A, either AGO2 or DICER looked like showing little change in the level over the course of heat treatment at 40°C by our Western blotting analyses, though a previous study reported an increase in the level of DICER under a heat-shocked condition (39.5°C) [25].

We also investigated the expression of endogenous genes carrying putative binding sites of miRNAs in their 3′-untranslated regions (3′UTRs): the *type IV alpha collagen* (*COL4A1* and *COL4A2* encoding α1 and α2 chain, respectively), *Laminin, gamma 1* (*LAMC1*), *DICER1*, *DNA methyltransferase 1* (*DNMT1*), *calcineurin binding protein 1* (*CABIN1*) and *GAPDH* genes were examined. RT-qPCR analyses using λpolyA^+^ RNA-A as an external control indicated that the expression level of *COL4A2*, *DICER1*, *LAMC1*, *DNMT1* and *GAPDH* was significantly decreased after heat shock; but *COL4A1* and *CABIN1* appeared to remain unchanged (Fig. S5). Since the apparent expression level of genes is likely dependent upon transcriptional activity and post-transcriptional regulation, the decrease in the level of *COL4A2*, *DICER1*, *LAMC1*, *DNMT1* and *GAPDH* after heat shock might reflect in part the enhancement of miRNA-mediated gene silencing, and the level of *COL4A1* and *CABIN1* might have been influenced by a strong transcriptional activity. Regarding a discrepancy between the *DICER1* mRNA level and its protein level, the stability of the protein may influence the results: the protein might be stable in some degree even under heat-stress conditions, and a slight difference in the level of the protein might be exhibited after heat-shock. But, such a difference could not be detected by our Western blot analysis.

With regard to alteration of the whole of gene expression after heat shock, our findings appeared to be consistent with the previous gene expression profile analysis indicating a major shift of mRNA population after heat shock, in which many genes were downregulated after heat shock [26]. Taken together, the mRNA level of many genes is presumably decreased under heat-stress conditions.

### Association of HSP90 with gene silencing

**Figure 3:**
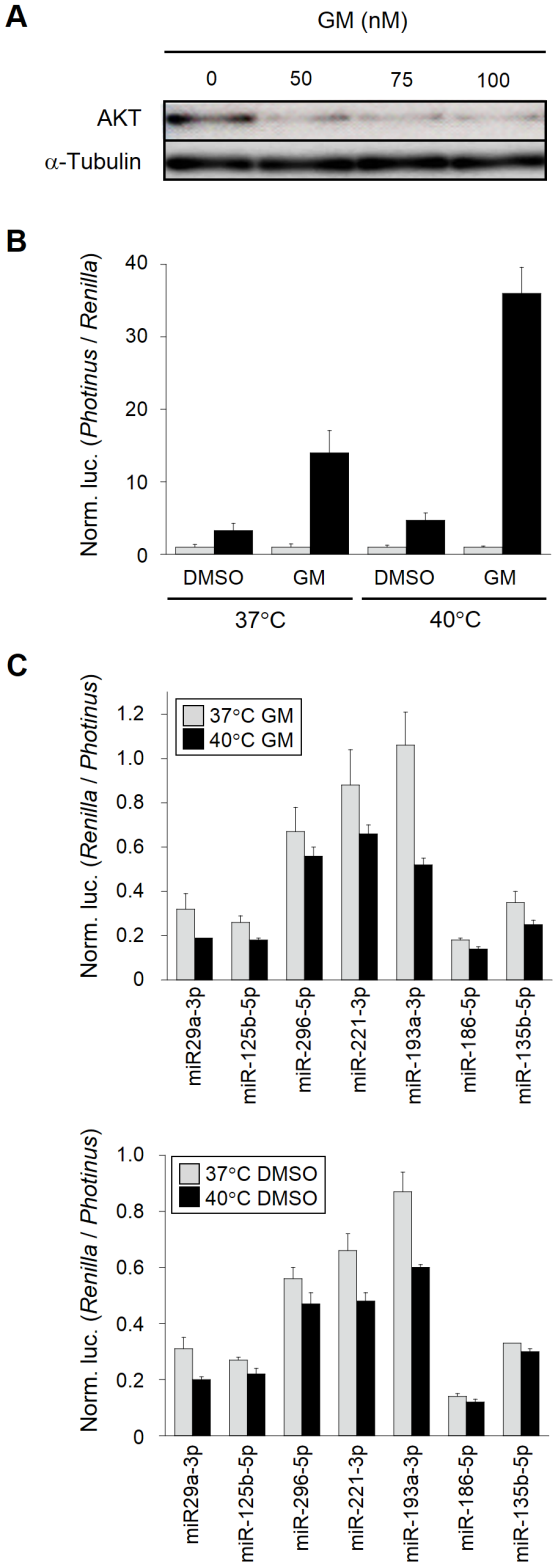
Effects of geldanamycin on gene silencing involving miRNAs. (**A**) Stability of AKT. To verify the efficacy of geldanamycin (GM) in HeLa cells, AKT that is a HSP90 client was examined in cells treated with various concentrations (nM) of geldanamycin by means of Western blotting. Alpha-tubulin was examined as an internal control. (**B**) Activation of HSF1 by GM. The pGL4-HSE (black bars) and pGL4-minP (gray bars; as a control) reporter plasmids were co-transfected with phRL-TK vector into HeLa cells. After 18 h-incubation, culture medium was changed to fresh medium containing 10 µM GM or DMSO (vehicle), and after 30 min a half of each treated cells were subjected to a mild hyperthermia at 40°C for 12 h. The expression levels of luciferase reporter genes were examined by a dual luciferase assay, and the activity of *Photinus* (target) luciferase was normalized to that of *Renilla* (control) luciferase, and further normalized to the data of pGL4-minP given as 1. The data are averages of three independent experiments and error bars represent standard deviations. (**C**) Gene silencing profiles in the presence of GM. The constructed reporter plasmids for assessment of gene silencing involving miRNAs were transfected into HeLa cells, and the cells were subjected to the same treatments as in B. The assessment of gene silencing was performed as in Fig. 1C. The data are averages of three independent experiments and error bars represent standard deviations. The upper and lower panels indicate the data in the presence and absence of GM, respectively.

Recent studies suggested that HSP90, which functions as a molecular chaperon, was involved in loading small RNA duplexes into AGO proteins in an ATP-dependent manner [23], and that HSP90-alpha, an isoform of HSP90, was upregulated by heat shock [27]. Accordingly, we examined whether HSP90 participated in changing in the suppression efficiency of miRNA-mediated gene silencing under heat-stress conditions. Gene silencing assay with reporter plasmids was carried out in heat-shocked HeLa cells in the presence and absence of geldanamycin that is a HSP90 inhibitor capable of binding to the ATP-binding domain of HSP90 [28]. To verify the inhibitory efficacy of geldanamycin on HSP90, first we investigated AKT and HSF1: AKT is a client protein of HSP90 and maintained stable by HSP90, and HSF1 is also associated with HSP90 under normal condition [21], [28]–[30]. Western blot analysis revealed that AKT was markedly decreased in the level after treatment with geldanamycin, indicating the instability of AKT by inhibition of HSP90 (Fig. 3A). The binding of geldanamycin to HSP90 is also conductive to dissociation of HSF1 from HSP90, thereby activating HSF1 [29], [30]. To examine the activation of HSF1, we introduced the pGL4-HSE reporter plasmid carrying the heat shock element (HSE) in the promoter region of the *Photinus luciferase* reporter gene into HeLa cells and treated the cells with and without geldanamycin. As shown in Fig. 3B, the geldanamycin treatment resulted in increase in the expression of the *luciferase* under either normal or mild hyperthermia conditions, and the expression of the reporter gene in the geldanamycin- plus heat-treated HeLa cells was markedly increased. All the data above indicated that the inhibition of HSP90 by geldanamycin had properly occurred in our treated cells. However, the gene silencing assay with and without the geldanamycin treatment exhibited similar results between them (Fig. 3C). Therefore, the findings suggested that HSP90 might hardly contribute to changing in the suppression efficiency of miRNA-mediated gene silencing under heat-stress conditions.

HSP90 is also known to be associated with cytoplasmic processing bodies (P-bodies) and stress granules, in which translationally inactivated mRNA and small RNAs including miRNAs appear to be sequestered [31]–[33]. Previous studies suggested that P-bodies themselves were not prerequisite for miRNA-mediated gene silencing, but that P-body formation was likely a consequence of gene silencing against mRNAs [33], [34]. Accordingly, the enhancement of miRNA-mediated gene silencing might contribute to acceleration of P-body formation under heat-stress conditions in a HSP90 independent manner.

### Effects of HSF1 knockdown on gene silencing involving miRNAs

**Figure 4:**
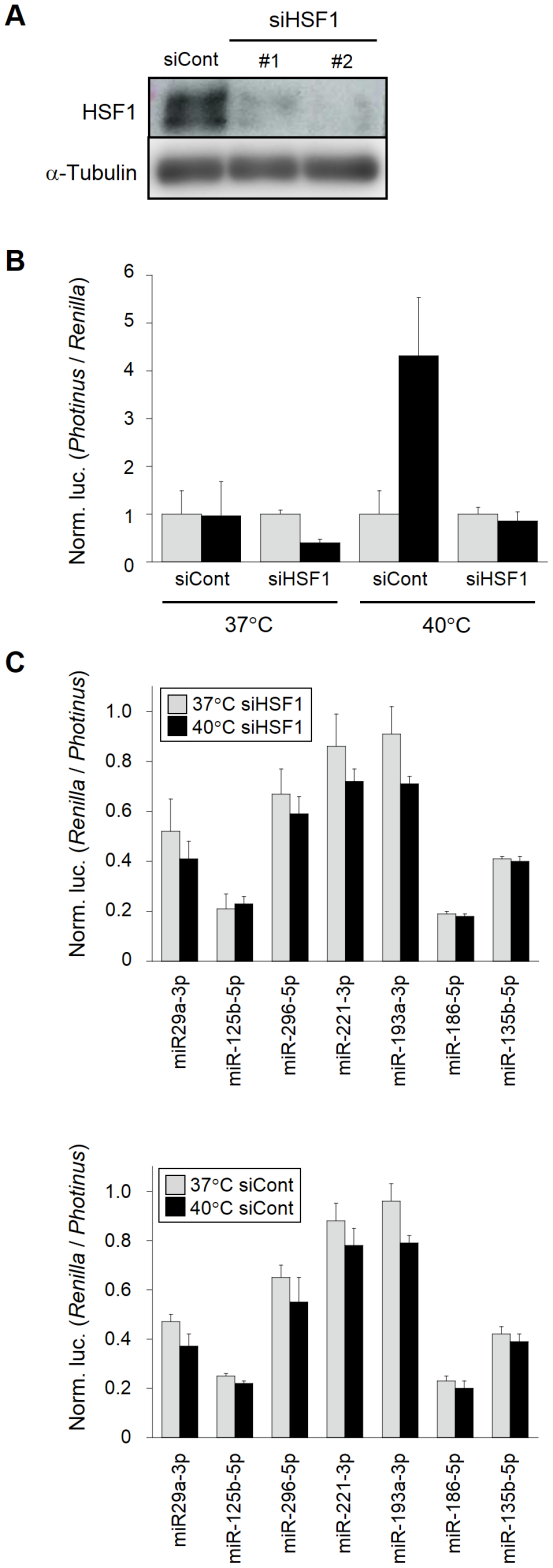
Gene silencing involving miRNAs under a *HSF1*-suppressed condition. (**A**) RNAi knockdown against *HSF1*. Two siRNAs directed against *HSF1* (siHSF1_#1 and #2) were designed, synthesized and introduced into HeLa cells. As a non-silencing control, siControl (siCont) was transfected into HeLa cells as well. 24 h after transfection, RNAi knockdown of HSF1 was examined by Western blotting, and alpha-tubulin was also examined as an internal control. (**B**) Effect of the knockdown of HSF1 on gene expression. HeLa cells were transfected with siHSF1_#2 and siControl, and after 48 h-incubation, the cells were subjected to the second transfection with pGL4-HSE (black bars) and pGL4-minP (gray bars) together with phRL-TK. After further 18 h-incubation, a half of each treated cells was subjected to a mild hyperthermia at 40°C for 12 h. The expressed luciferase reporters were examined and analyzed by using the data obtained with siControl as a reference, as in Fig. 3B. (**C**) Gene silencing profiles in HSF1-silenced cells. The same experiments as in B except for the reporter plasmids, which were all swapped for the reporter plasmids indicated in Fig. 3C, were carried out. The assessment of gene silencing involving endogenous miRNAs was performed as in Fig. 1C. The data are averages of three independent experiments and error bars represent standard deviations. The upper and lower panels indicate the gene silencing profiles with and without knockdown of HSF1, respectively.

Heat shock stress most likely stimulates (activates) various vital pathways including heat shock responses triggered by HSF1 [20]–[22]. HSF1 is known to be phosphorylated as an immediate response to heat shock, and phosphorylated HSF1 is capable of binding to HSEs in the promoters of HSP genes, *i.e.*, HSF1 likely works as an initial triggering molecule in heat shock responses. Accordingly, we investigated miRNA-mediated gene silencing under a HSF1-suppressed condition by RNAi. We designed siRNAs against *HSF1* and the siRNAs could confer a strong inhibition of HSF1 (Fig. 4A). In addition, the expression of the *luciferase* reporter gene linked with HSE was consistently suppressed in the presence of our designed siRNAs (Fig. 4B). Under such a HSF1-suppressed condition, the activity of gene silencing involving miRNAs was examined, and the results displayed similar profiles of the gene silencing activity between the HSF1-knockdown and naïve HeLa cells (Fig. 4C). Therefore, it was suggested that HSF1 might hardly participate in changing the suppression efficiency of miRNA-mediated gene silencing in heat-stressed cells.

### Influence of the inhibition of signal pathways on gene silencing

Not only HSF1 but also other factors that participate in vital signal pathways may be activated by heat shock [22], [35]–[38]. In fact, our findings indicated that the reporter genes carrying either the cAMP response element (CRE), serum response factor response element (SRF-RE), or serum response element (SRE) [39]–[41] as well as HSE were activated by heat shock (Fig. S6). In addition, recent studies suggested that signal pathways involving PI3K/AKT and MAPK participated in the posttranslational modification of AGO2 [42]–[44], leading to the possibility that PI3K/AKT and MAPK might be involved in gene silencing. We investigated miRNA-mediated gene silencing in the presence of protein kinase inhibitors directed to PI3K, MEK and p38/MAPK. As shown in Fig. S7, the results indicated that the examined protein kinase inhibitors, like geldanamycin and siHSF1, hardly influenced the gene silencing profiles mediated by miRNAs in either heat-treated or naïve HeLa cells, suggesting that the signal pathways involving PI3K, MEK and p38/MAPK might not be involved in changing the suppression efficiency of miRNA-mediated gene silencing in heat-shocked cells.

## Discussion

Sudden change in temperature, or heat shock, is a substantial stress to cells, and cells immediately respond to such a stress for protecting themselves and for maintenance of homeostasis. Heat-denaturation of proteins is a major harmful occurrence in heat-shocked cells. To restore such denatured proteins to normal proteins and to mitigate the harmful state of cells, heat-shocked cells immediately activate and express HSPs that function as a molecular chaperon. HSPs appear to play important roles in the posttranslational restoration and/or maintenance of proteins under heat stress conditions [19]–[22]. Another possible response to heat stress is presumably the suppression of expression except for the expression of *HSPs*, thereby possibly leading to the reduction of heat-denatured proteins in heat-shocked cells. The previous study [26] and our data (Fig. S5) consistently showed that many genes were downregulated after heat shock. In this study, we focused on the gene silencing mediated by endogenous miRNAs as a potential contributor to mitigate the harmful gene products caused by heat stress or heat denaturation, and our findings suggested that the activity of the gene silencing was enhanced in mammalian cells which exposed to a mild hyperthermia. The enhancement of the gene silencing presumably makes sense in heat-shocked cells where the emergence of misfolded proteins or heat-denatured proteins were aggressively suppressed. Because the gene silencing involving miRNAs is a post-transcriptional inhibition, or a pre-translational inhibition, the enhancement of the gene silencing under heat-stress conditions may help the suppression of the emergence of heat-denatured proteins via its aggressive inhibition of translation. Therefore, we propose that the gene silencing mediated by miRNAs, together with HSP chaperons, likely participate in the protection of cells from harmful heat-stress conditions by engaging in the prevention of the emergence of heat-denatured proteins in heat-shocked cells.

It is of interest to understand the mechanism of the enhancement of miRNA-mediated gene silencing in heat-shocked cells. Our current findings suggested that the enhancement of the gene silencing was probably independent of the cytoprotective action involving HSPs, though the recent study indicated that either HSP70 or HSP90 would be associated with AGO2, which is a key component of RISC, and involved in loading small RNA duplexes into RISCs [23]. Additionally, our data exhibited that no significant decrease nor increase in either AGO2 or DICER occurred after heat treatment. The previous gene expression analyses in heat-treated and untreated *Hsf1*-deficient mouse embryonic fibroblasts revealed that certain clusters of genes were upregulated after heat shock even in the absence of *Hsf1* [45], and genes related to gene silencing such as *Ago2* and *Dicer* were absent from the clusters of genes. Accordingly, the enhancement of miRNA-mediated gene silencing might not be caused by change in the expression of gene silencing-related genes.

AGO2 is known to be phosphorylated. AKT, MAPK-activated protein kinase-II (MKII), and epidermal growth factor receptor (EGFR) appeared to be involved in the phosphorylation of AGO2 [42], [43], [46]; and previous studies indicated that AKT and MKII were activated by heat shock [35]–[37]. Our experiments consistently showed that the reporter genes carrying SRE, SRF-RE and CRE as well as HSE were activated by a mild hyperthermia (Fig. S6), *i.e.*, heat shock activated signaling pathways. Based on the findings, we performed inhibition assays with various inhibitors including protein kinase inhibitors; however, little or no influence of the inhibitors on the enhancement of the miRNA-mediated gene silencing under heat-stress conditions was observed in our current study.

From the findings presented here, we speculated possible mechanisms for the enhancement of the miRNA-mediated gene silencing under heat-stress conditions: (i) superfluous thermal energy by heat shock might cause a conformational change in AGO2 (or RISC as a whole) and such a conformational change might facilitate efficient scanning of target mRNAs as well as loading of miRNAs to RISCs in a HSP90 independent manner, thereby inducing the enhancement of the miRNA-mediated gene silencing; and alternatively (ii) unknown factor(s) which was unaffected by the inhibitors used in this study might be involved in the enhancement triggered by heat shock. In either case, more extensive studies need to be carried out to elucidate the mechanism(s) of the enhancement of miRNA-mediated gene silencing under heat-stress conditions.

It should be noted that heat-shocked cells appeared to possess miRNAs that conferred unchanged gene silencing and the reduction of gene silencing, as well as miRNAs associated with the enhancement of gene silencing; *i.e.*, at least three sorts of miRNAs, which are involved in unchanged, weakened and enhanced gene silencing, may exist in heat-shocked cells. Such differences in miRNAs might be caused by (or dependent upon) the sequences of miRNAs themselves. On a relevant note, different small interfering RNAs (siRNAs), which are equal to miRNAs, are capable of inducing different levels of RNAi activity in mammalian cells [47], [48], *i.e.*, siRNA-dependent gene silencing in mammalian cells. Therefore, miRNA-dependent regulation of gene silencing might occur in heat-shocked cells as well. To further clarify the difference between miRNAs conferring enhanced gene silencing and unchanged gene silencing in heat-shocked cells as well as to elucidate the mechanism of the enhancement of miRNA-mediated gene silencing, more extensive studies need to be carried out in the future.

Finally, our current study may shed light on a new contribution of gene silencing mediated by endogenous miRNAs to aggressive suppression of the emergence of heat-denatured proteins in heat-shocked cells, and also provide a new insight into the cytoprotective action against heat-stress environment.

## Supporting Information

Figure S1 **Schematic drawing of constructed reporter plasmid.** The reporter plasmids were constructed with the psiCHECK-2 vector by inserting synthetic oligonucleotide duplexes directed to miRNAs of interest into the 3′-untranslated region (3′-UTR) of the *Renilla luciferase* gene. The sequences of miRNAs (indicated by N) in the synthetic oligoDNAs are shown in Table S1. The inserted oligonucleotide duplexes carry the *Spe* I restriction enzyme site for judgment of proper clones, and possess a cohesive and a blunt ends matched to the *Xho* I and *Pme* I digested ends, respectively. The SV40 and TK promoters and *Photinus* luciferase as a control reporter gene are indicated. CDR: coding region. When the reporter plasmids are introduced into mammalian cells, both the reporter genes in the plasmids are expressed. If miRNAs function in cells (in RISCs), the *Renilla luciferase* transcripts carrying miRNA-complementary sequences in their 3′-UTRs will become targets for the miRNAs in gene silencing, resulting in suppression of the *Renilla* luciferase catalytic activity. The *Photinus luciferase* transcript is a non-target RNA of the miRNAs and capable of becoming a control for normalization of the target *Renilla luciferase*.
(TIF)

Figure S2 **Gene silencing profile involving endogenous miRNAs.** The data of gene silencing that were obtained with 143 constructed reporter plasmids were arranged in increasing order of the normalized luciferase expression ratios at 37°C, and aligned from the lowest value (left) to the highest one (right). The data obtained at 37°C (blue bars) were overlapped with the data that were obtained at 40°C (yellow bars), in which the data showing a statistically significant decrease (*P*<0.05) were indicated by red bars.
(TIF)

Figure S3 **Expression levels of** ***luciferase*** **reporter genes under a mild hyperthermia.** HeLa cells were transfected with psiCHECK-2 empty vector. 18 h after transfection, the cells were exposed to a mild hyperthermia at 40°C for 12 h. Cell extract was prepared and subjected to determination of protein concentration by a protein assay kit. The expression of the *Photinus* and *Renilla* luciferase genes was examined by a dual luciferase assay. The activities of the *Renilla* and *Photinus* luciferases were normalized to the protein concentration. Data are averages of three independent experiments and error bars represent standard deviations. The data are indicated by arbitrary units (A.U.).
(TIF)

Figure S4 **Analyses of miR-494.** (**A**) Expression of miR-494 in heat-shocked cells. HeLa cells were treated as in Fig. 2. The expression of primary miR-494 (Pri-miR-494) and matured miR-494 (miR-494) was examined by RT-qPCR. The expression data were analyzed as in Fig. 2B. (**B**) Gene silencing activity involving miR-494. A psiCHECK-2 backbone plasmid that carried the complementary sequence of miR-494 was constructed (psiCHECK-miR-494), and transfected into HeLa cells followed by a mild hyperthermia as in Fig. 1C. The obtained data were analyzed as in Fig. 1C (n = 3; mean ± SDs).
(TIF)

Figure S5 **Expression profile of endogenous genes.** Examined genes are indicated: *COL4A1*, *COL4A2*, *DICER1*, *LAMC1*, *DNMT1*, *CABIN1* and *GAPDH*. After heat treatment as in Fig. 2, total RNAs were isolated from cell extracts containing λpolyA^+^ RNA-A as an external control, and examined by RT-qPCR followed by analysis using delta-delta Ct method with the Ct of λpolyA^+^ RNA-A as a control. The data were further normalized to the data obtained at 37°C as 1 (n = 3; mean ± SDs). Statistical analysis was carried out by Student's *t*-test (two-tailed): asterisks represent significant decreases versus the 37°C data (*p*<0.05).
(TIF)

Figure S6 **Promoter activities under a mild hyperthermia.** The pGL4 vectors that encode various response elements in the minimum promoter of the *Photinus luciferase* reporter gene were co-transfected with the phRL-TK vector carrying *Renilla luciferase* as a control into HeLa cells. 18 h after transfection, the cells were subjected to a mild hyperthermia at 40°C for 6 h or 12 h, followed by a dual luciferase assay as in Fig. 1C. The activity of the *Photinus* luciferase was normalized to that of the *Renilla* luciferase, and further normalized to the data obtained from the cells that were transfected with the pGL4 empty vector (minP) and incubated at 37°C. Data are averages of three independent experiments and error bars represent standard deviations. The response elements investigated were as follows: Heat shock element, HSE; Serum response element, SRE; Serum response factor response element, SRF-RE; cyclic AMP response element, CRE; Interferon-stimulated response element, ISRE; Nuclear factor κB response element, NF-κB-RE.
(TIF)

Figure S7 **Effects of kinase inhibitors on gene silencing involving miRNAs.** HeLa cells that were transfected with the reporter plasmids as in Fig. 3 were treated with PI3K-, MEK-, p38/MAPK-inhibitors and DMSO (vehicle) as a control for 30 min, followed by a mild hyperthermia (at 40°C for 12 h) and a dual luciferase assay as in Fig. 3. The obtained data were analyzed as in Fig. 3. Data are averages of four independent experiments and error bars represent standard deviations.
(TIF)

Table S1 **Oligo DNA sequences directed against miRNAs.** The N-sequences of the ssOligo DNA shown in Fig. S1 are indicated, and the sequences are complementary to indicated miRNAs. Regarding let-7-3xbulge, the reporter plasmid carrying the let-7-3xbulge sequence was constructed previously [24] and used in this study.
(XLSX)
