# Supplementary figures and images for: Gene Silencing Mediated by Endogenous MicroRNAs under Heat Stress Conditions in Mammalian Cells
Source: PLoS One. 2014 Jul 28;9(7):e103130. doi: 10.1371/journal.pone.0103130 (PMC4113354; doi:10.1371/journal.pone.0103130)

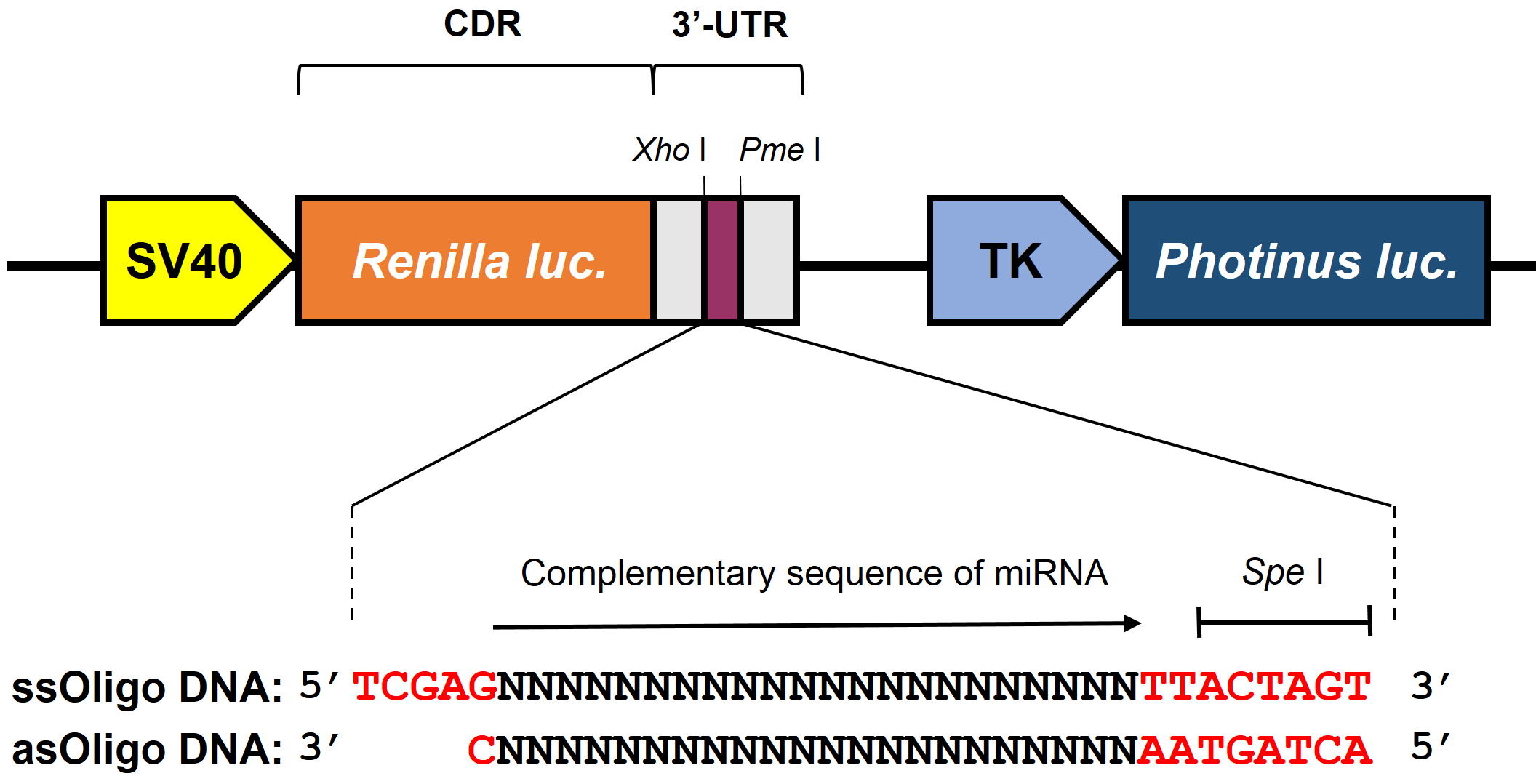

Supplement: Figure S1 — Schematic drawing of constructed reporter plasmid. The reporter plasmids were constructed with the psiCHECK-2 vector by inserting synthetic oligonucleotide duplexes directed to miRNAs of interest into the 3′-untranslated region (3′-UTR) of the Renilla luciferase gene. The sequences of miRNAs (indicated by N) in the synthetic oligoDNAs are shown in Table S1. The inserted oligonucleotide duplexes carry the Spe I restriction enzyme site for judgment of proper clones, and possess a cohesive and a blunt ends matched to the Xho I and Pme I digested ends, respectively. The SV40 and TK promoters and Photinus luciferase as a control reporter gene are indicated. CDR: coding region. When the reporter plasmids are introduced into mammalian cells, both the reporter genes in the plasmids are expressed. If miRNAs function in cells (in RISCs), the Renilla luciferase transcripts carrying miRNA-complementary sequences in their 3′-UTRs will become targets for the miRNAs in gene silencing, resulting in suppression of the Renilla luciferase catalytic activity. The Photinus luciferase transcript is a non-target RNA of the miRNAs and capable of becoming a control for normalization of the target Renilla luciferase. (TIF) [file pone.0103130.s001.tif]

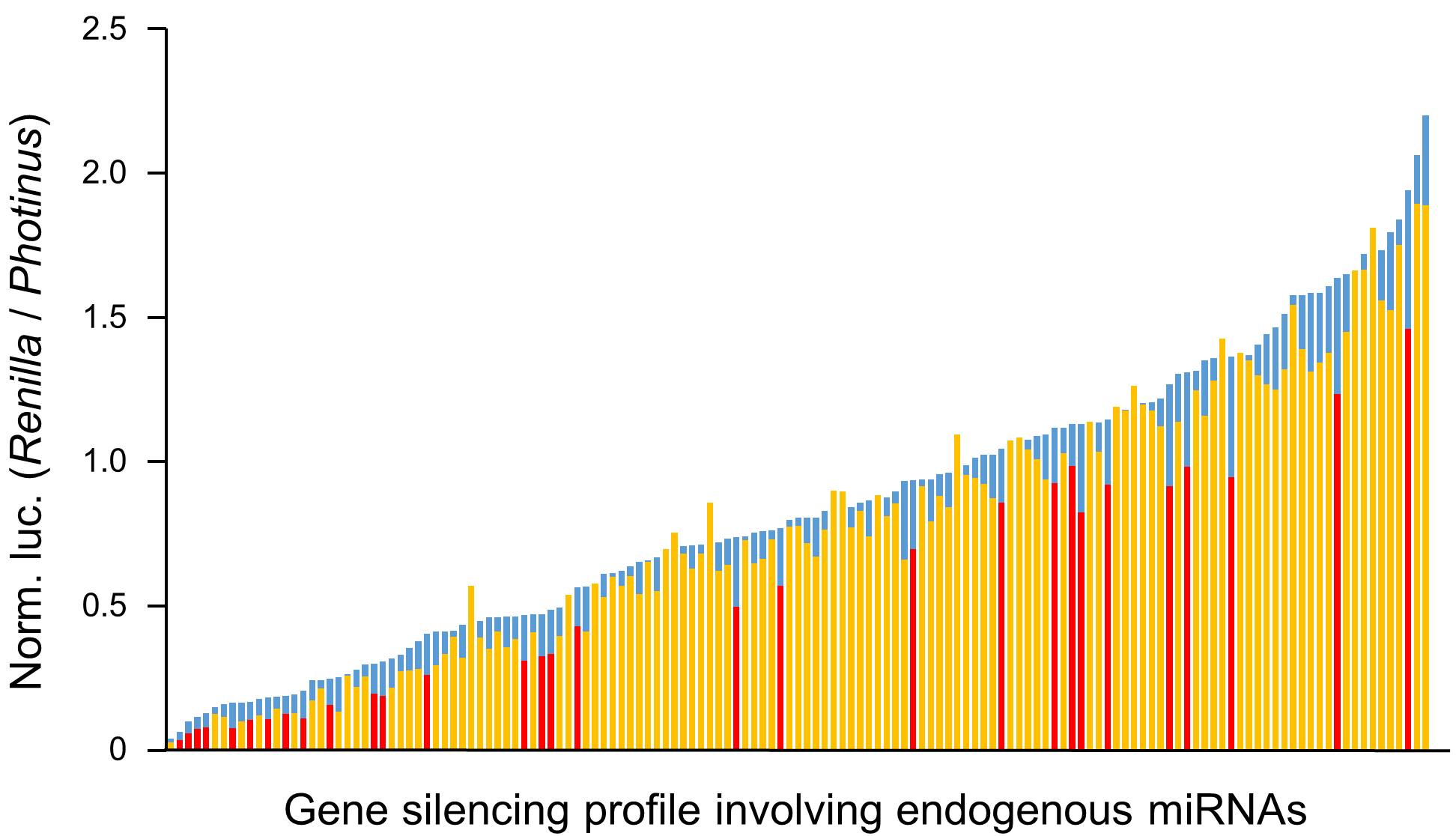

Supplement: Figure S2 — Gene silencing profile involving endogenous miRNAs. The data of gene silencing that were obtained with 143 constructed reporter plasmids were arranged in increasing order of the normalized luciferase expression ratios at 37°C, and aligned from the lowest value (left) to the highest one (right). The data obtained at 37°C (blue bars) were overlapped with the data that were obtained at 40°C (yellow bars), in which the data showing a statistically significant decrease (P<0.05) were indicated by red bars. (TIF) [file pone.0103130.s002.tif]

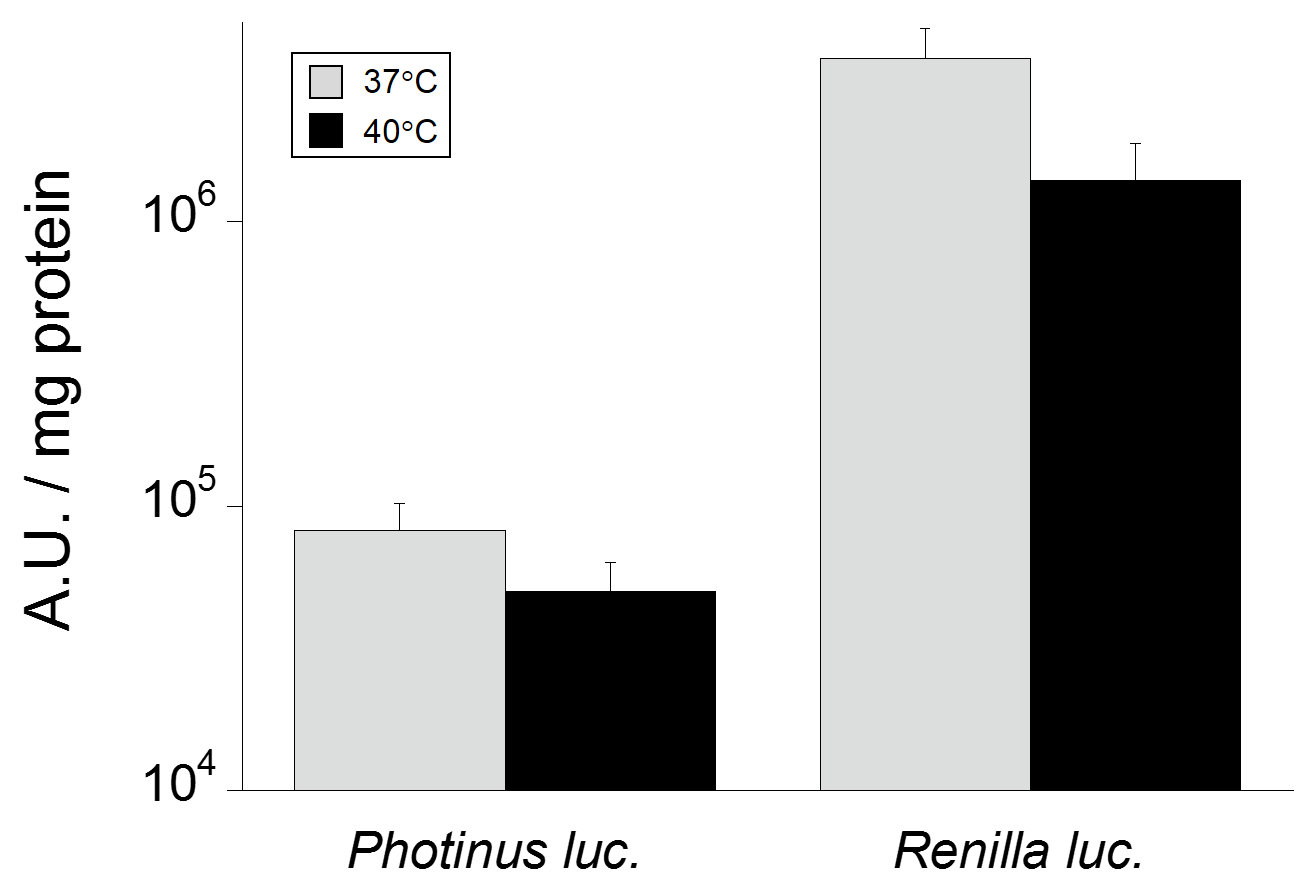

Supplement: Figure S3 — Expression levels of luciferase reporter genes under a mild hyperthermia. HeLa cells were transfected with psiCHECK-2 empty vector. 18 h after transfection, the cells were exposed to a mild hyperthermia at 40°C for 12 h. Cell extract was prepared and subjected to determination of protein concentration by a protein assay kit. The expression of the Photinus and Renilla luciferase genes was examined by a dual luciferase assay. The activities of the Renilla and Photinus luciferases were normalized to the protein concentration. Data are averages of three independent experiments and error bars represent standard deviations. The data are indicated by arbitrary units (A.U.). (TIF) [file pone.0103130.s003.tif]

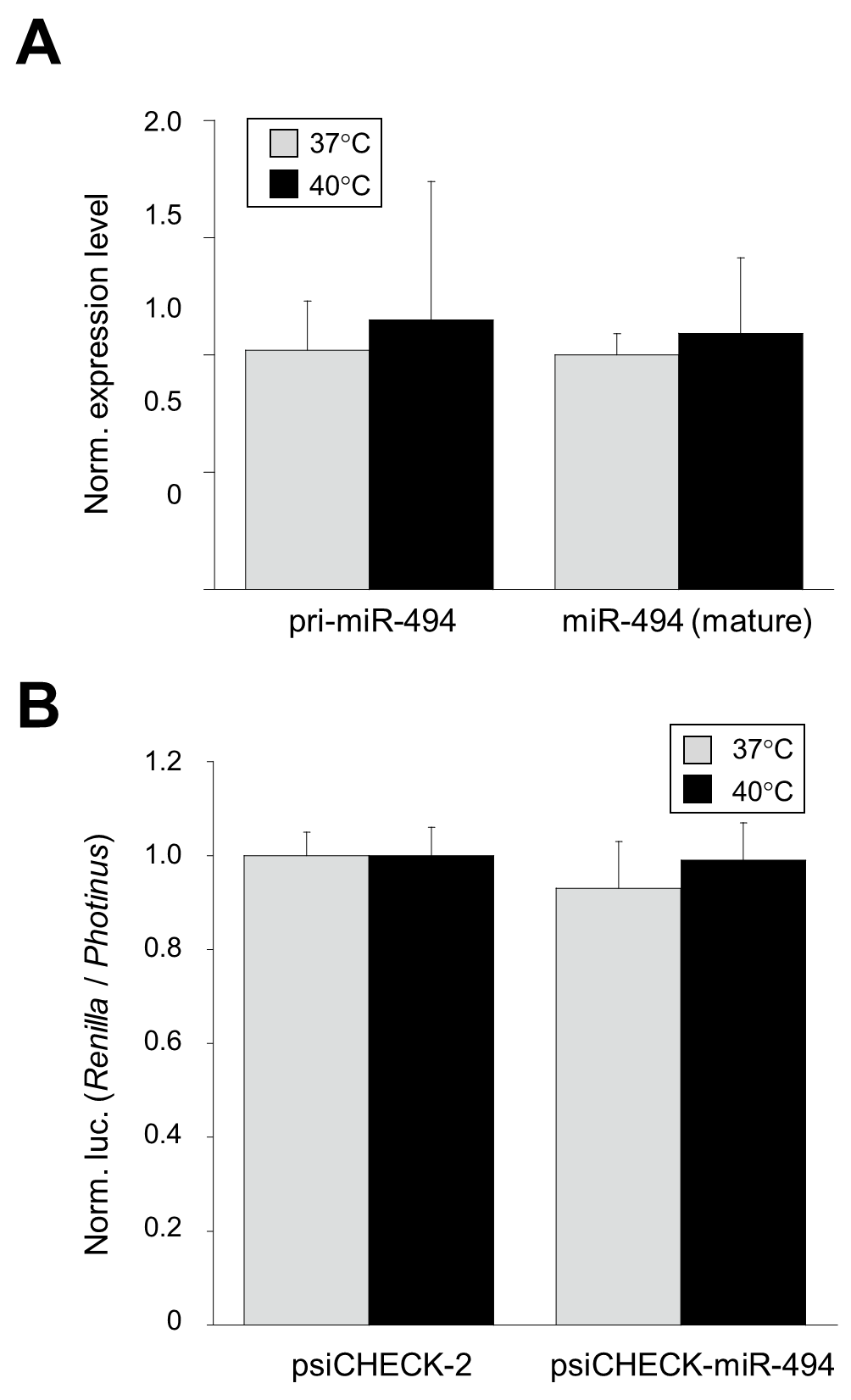

Supplement: Figure S4 — Analyses of miR-494. (A) Expression of miR-494 in heat-shocked cells. HeLa cells were treated as in Fig. 2. The expression of primary miR-494 (Pri-miR-494) and matured miR-494 (miR-494) was examined by RT-qPCR. The expression data were analyzed as in Fig. 2B. (B) Gene silencing activity involving miR-494. A psiCHECK-2 backbone plasmid that carried the complementary sequence of miR-494 was constructed (psiCHECK-miR-494), and transfected into HeLa cells followed by a mild hyperthermia as in Fig. 1C. The obtained data were analyzed as in Fig. 1C (n = 3; mean ± SDs). (TIF) [file pone.0103130.s004.tif]

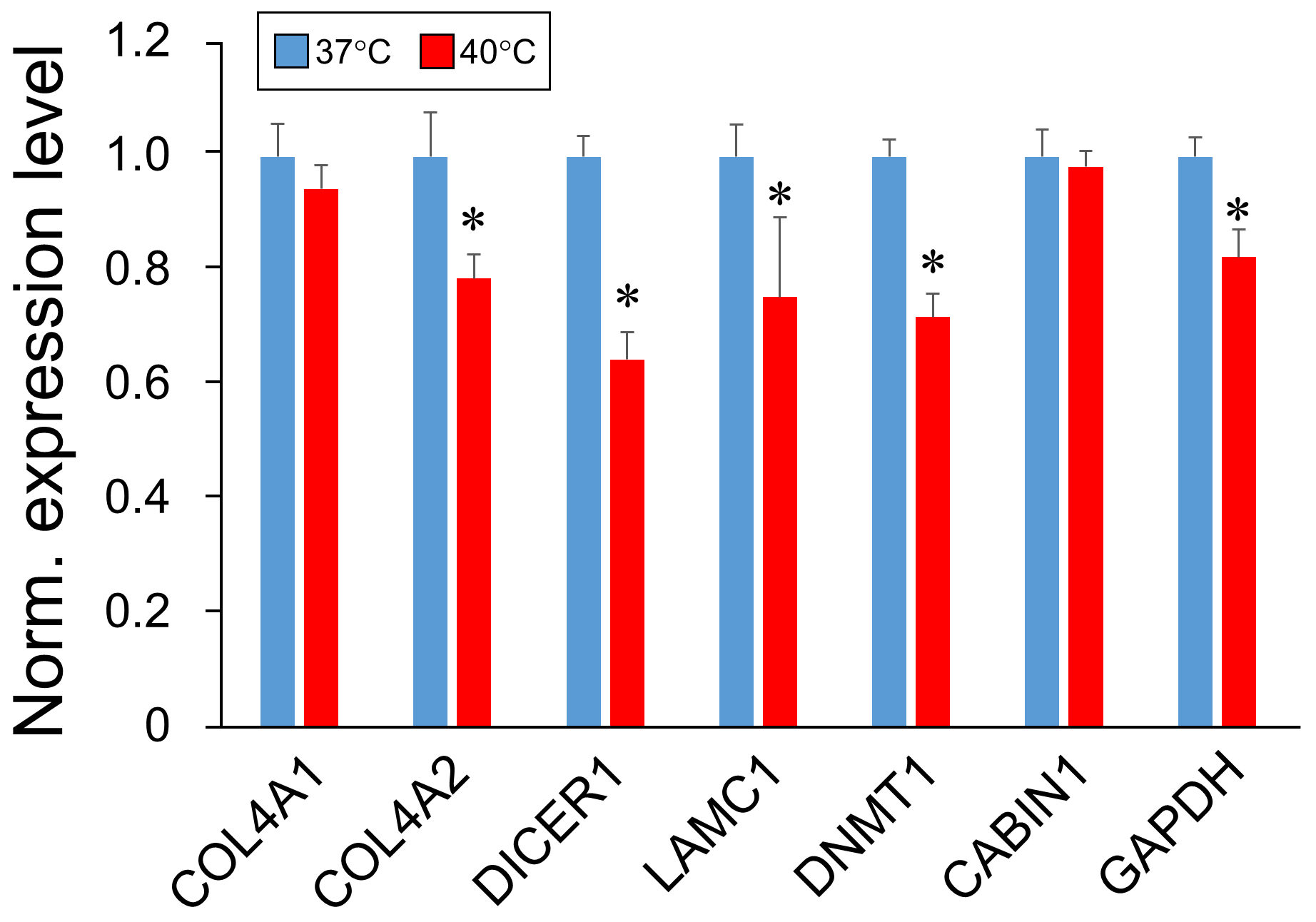

Supplement: Figure S5 — Expression profile of endogenous genes. Examined genes are indicated: COL4A1, COL4A2, DICER1, LAMC1, DNMT1, CABIN1 and GAPDH. After heat treatment as in Fig. 2, total RNAs were isolated from cell extracts containing λpolyA+ RNA-A as an external control, and examined by RT-qPCR followed by analysis using delta-delta Ct method with the Ct of λpolyA+ RNA-A as a control. The data were further normalized to the data obtained at 37°C as 1 (n = 3; mean ± SDs). Statistical analysis was carried out by Student's t-test (two-tailed): asterisks represent significant decreases versus the 37°C data (p<0.05). (TIF) [file pone.0103130.s005.tif]

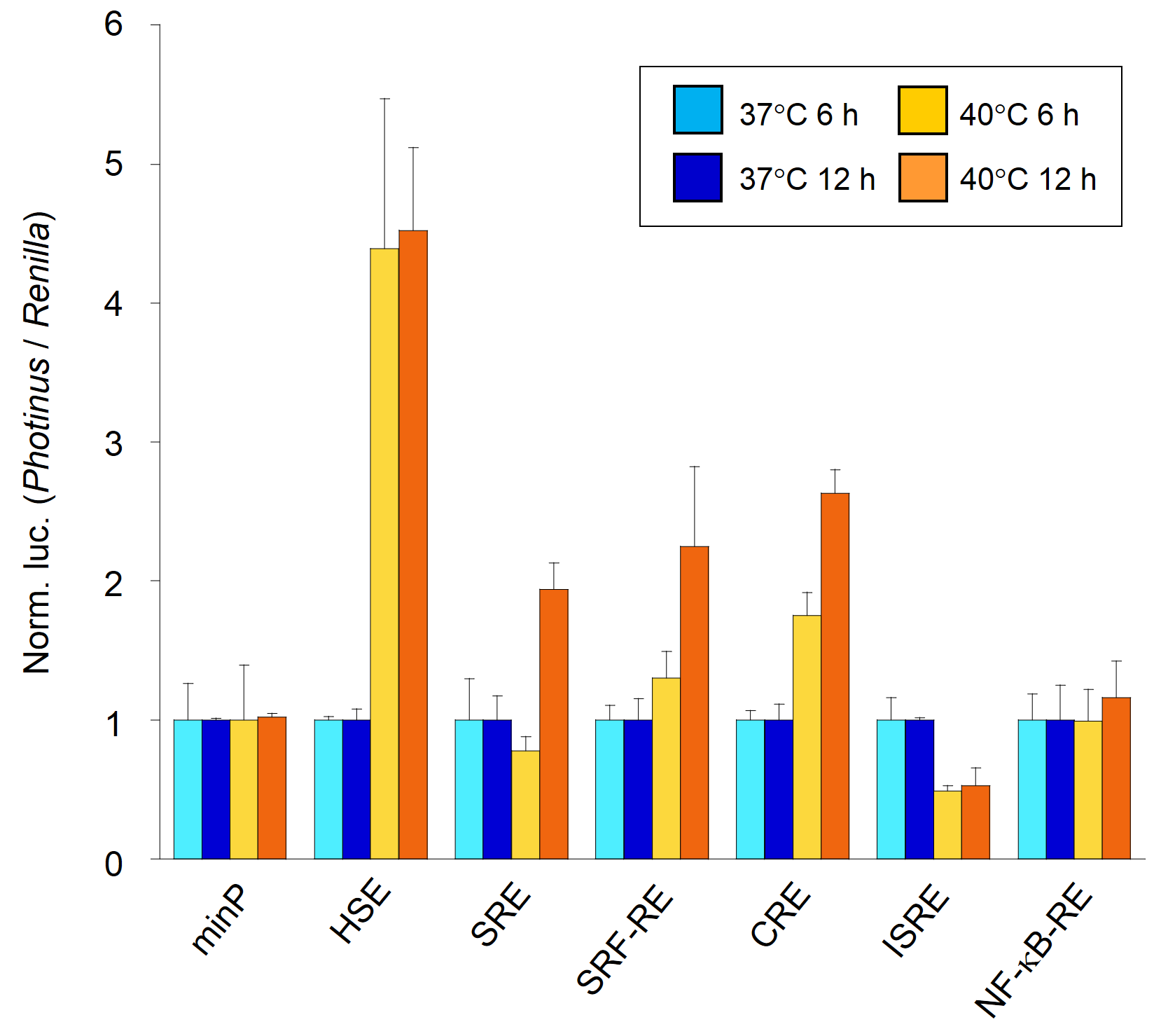

Supplement: Figure S6 — Promoter activities under a mild hyperthermia. The pGL4 vectors that encode various response elements in the minimum promoter of the Photinus luciferase reporter gene were co-transfected with the phRL-TK vector carrying Renilla luciferase as a control into HeLa cells. 18 h after transfection, the cells were subjected to a mild hyperthermia at 40°C for 6 h or 12 h, followed by a dual luciferase assay as in Fig. 1C. The activity of the Photinus luciferase was normalized to that of the Renilla luciferase, and further normalized to the data obtained from the cells that were transfected with the pGL4 empty vector (minP) and incubated at 37°C. Data are averages of three independent experiments and error bars represent standard deviations. The response elements investigated were as follows: Heat shock element, HSE; Serum response element, SRE; Serum response factor response element, SRF-RE; cyclic AMP response element, CRE; Interferon-stimulated response element, ISRE; Nuclear factor κB response element, NF-κB-RE. (TIF) [file pone.0103130.s006.tif]

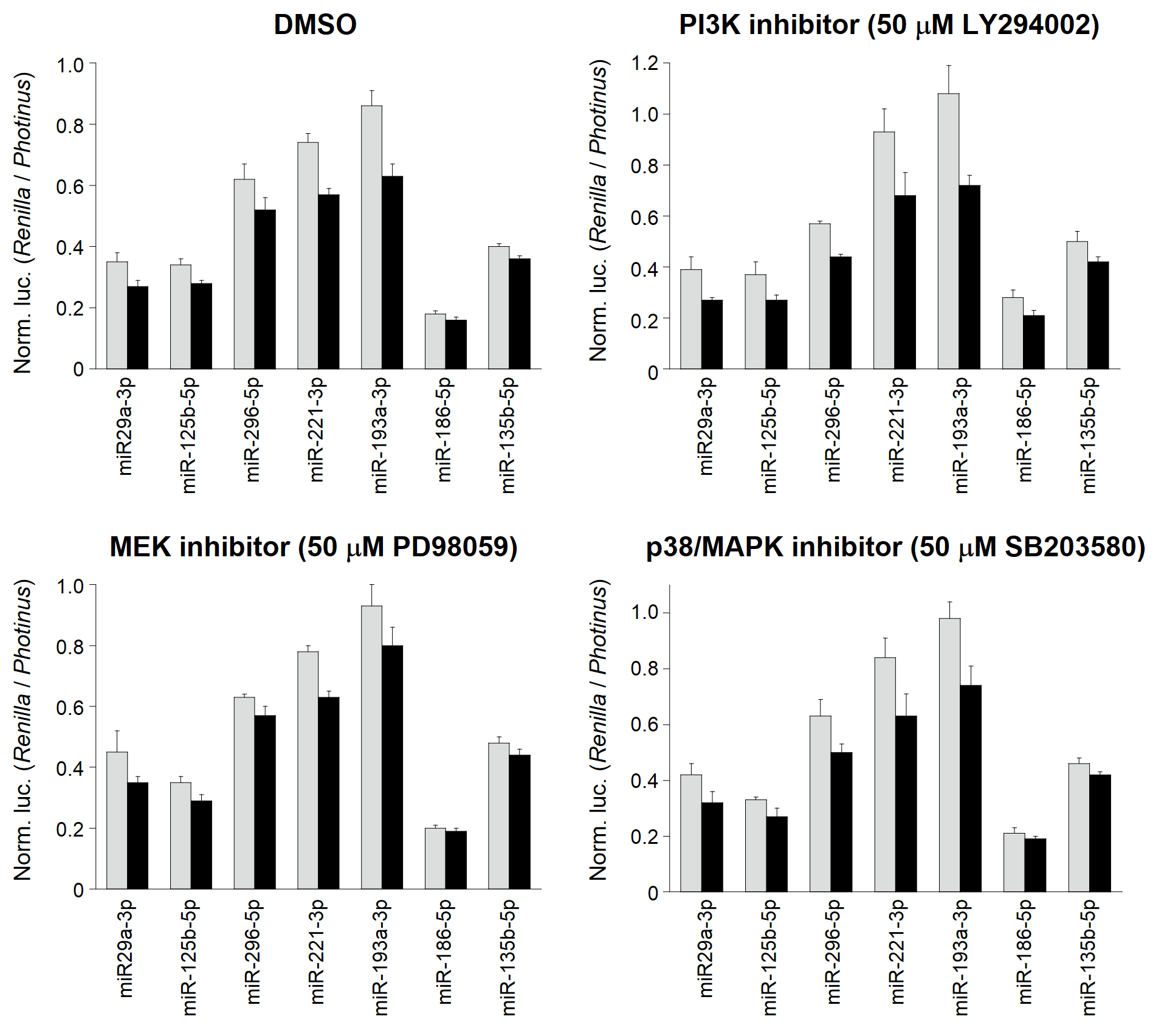

Supplement: Figure S7 — Effects of kinase inhibitors on gene silencing involving miRNAs. HeLa cells that were transfected with the reporter plasmids as in Fig. 3 were treated with PI3K-, MEK-, p38/MAPK-inhibitors and DMSO (vehicle) as a control for 30 min, followed by a mild hyperthermia (at 40°C for 12 h) and a dual luciferase assay as in Fig. 3. The obtained data were analyzed as in Fig. 3. Data are averages of four independent experiments and error bars represent standard deviations. (TIF) [file pone.0103130.s007.tif]
